# Low-Attenuation Noncalcified Plaque on Coronary Computed Tomography Angiography Predicts Myocardial Infarction: Results From the Multicenter SCOT-HEART Trial (Scottish Computed Tomography of the HEART)

**DOI:** 10.1161/CIRCULATIONAHA.119.044720

**Published:** 2020-03-16

**Authors:** Michelle C. Williams, Jacek Kwiecinski, Mhairi Doris, Priscilla McElhinney, Michelle S. D’Souza, Sebastien Cadet, Philip D. Adamson, Alastair J. Moss, Shirjel Alam, Amanda Hunter, Anoop S.V. Shah, Nicholas L. Mills, Tania Pawade, Chengjia Wang, Jonathan Weir McCall, Michael Bonnici-Mallia, Christopher Murrills, Giles Roditi, Edwin J.R. van Beek, Leslee J. Shaw, Edward D. Nicol, Daniel S. Berman, Piotr J. Slomka, David E. Newby, Marc R. Dweck, Damini Dey

**Affiliations:** 1 University/BHF Centre for Cardiovascular Science (M.C.W., J.K., M.D., M.S.D’S., P.D.A., A.J.M., S.A., A.H., A.S.V.S., N.L.M., T.P., C.W., E.J.R.v.B., D.E.N., M.R.D.), University of Edinburgh, United Kingdom.; 2 Edinburgh Imaging Facility QMRI (M.C.W., E.J.R.v.B., D.E.N., M.R.D.), University of Edinburgh, United Kingdom.; 3 Department of Interventional Cardiology and Angiology, Institute of Cardiology, Warsaw, Poland (J.K.).; 4 Cedars-Sinai Medical Centre, Los Angeles, CA (P.M., S.C., P.J.S., D.S.B., D.D.).; 5 University of Cambridge, United Kingdom (J.W.M.).; 6 Department of Radiology, Ninewells Hospital, Dundee, United Kingdom (M.B-M., C.M.).; 7 Institute of Clinical Sciences, University of Glasgow, United Kingdom (G.R.).; 8 Weill Cornell Medical College, New York, NY (L.J.S.).; 9 Royal Brompton and Harefield NHS Foundation Trust Departments of Cardiology and Radiology; and the National Heart and Lung Institute, Faculty of Medicine, Imperial College, London, United Kingdom (E.D.N.).; 10 Christchurch Heart Institute, University of Otago, Christchurch, New Zealand (P.D.A).

**Keywords:** atherosclerosis, cardiovascular diseases, coronary artery disease, computed tomography angiography, myocardial infarction, plaque, atherosclerotic

## Abstract

**Background:**

The future risk of myocardial infarction is commonly assessed using cardiovascular risk scores, coronary artery calcium score, or coronary artery stenosis severity. We assessed whether noncalcified low-attenuation plaque burden on coronary CT angiography (CCTA) might be a better predictor of the future risk of myocardial infarction.

**Methods:**

In a post hoc analysis of a multicenter randomized controlled trial of CCTA in patients with stable chest pain, we investigated the association between the future risk of fatal or nonfatal myocardial infarction and low-attenuation plaque burden (% plaque to vessel volume), cardiovascular risk score, coronary artery calcium score or obstructive coronary artery stenoses.

**Results:**

In 1769 patients (56% male; 58±10 years) followed up for a median 4.7 (interquartile interval, 4.0–5.7) years, low-attenuation plaque burden correlated weakly with cardiovascular risk score (*r*=0.34; *P*<0.001), strongly with coronary artery calcium score (*r*=0.62; *P*<0.001), and very strongly with the severity of luminal coronary stenosis (area stenosis, *r*=0.83; *P*<0.001). Low-attenuation plaque burden (7.5% [4.8–9.2] versus 4.1% [0–6.8]; *P*<0.001), coronary artery calcium score (336 [62–1064] versus 19 [0–217] Agatston units; *P*<0.001), and the presence of obstructive coronary artery disease (54% versus 25%; *P*<0.001) were all higher in the 41 patients who had fatal or nonfatal myocardial infarction. Low-attenuation plaque burden was the strongest predictor of myocardial infarction (adjusted hazard ratio, 1.60 (95% CI, 1.10–2.34) per doubling; *P*=0.014), irrespective of cardiovascular risk score, coronary artery calcium score, or coronary artery area stenosis. Patients with low-attenuation plaque burden greater than 4% were nearly 5 times more likely to have subsequent myocardial infarction (hazard ratio, 4.65; 95% CI, 2.06–10.5; *P*<0.001).

**Conclusions:**

In patients presenting with stable chest pain, low-attenuation plaque burden is the strongest predictor of fatal or nonfatal myocardial infarction. These findings challenge the current perception of the supremacy of current classical risk predictors for myocardial infarction, including stenosis severity.

**Registration:**

URL: https://www.clinicaltrials.gov; Unique identifier: NCT01149590.

## Abstract 2

Supplemental Digital Content is available in the text.

**Clinical Perspective**

**What Is New?**

- Quantitative assessment of atherosclerotic plaque on coronary computed tomography angiography can be used to measure the burden of calcified, noncalcified, and low-attenuation plaque, as well as the total coronary plaque burden, providing important prognostic information.
- In this study, low-attenuation plaque was the strongest predictor of fatal or nonfatal myocardial infarction, exceeding other established markers including cardiovascular risk scores, computed tomography calcium scoring, and coronary artery stenoses.
- Patients with a low-attenuation plaque burden >4% were 5 times more likely to suffer a fatal or nonfatal myocardial infarction.

**What Are the Clinical Implications?**

- The burden of low-attenuation coronary plaque can be measured semiautomatically on standard coronary computed tomography angiography scans, with the potential for widespread clinical adoption.
- This method provides incremental prediction of myocardial infarction to standard assessments of cardiovascular risk scores, computed tomography calcium scoring or luminal stenosis severity.
- Further studies should investigate whether this novel approach can used to guide clinical decision making and improve patient outcomes.

The current clinical paradigm of coronary artery disease is based on cardiovascular risk scores and the presence of obstructive stenoses in the coronary vasculature.^1,2^ Coronary computed tomography angiography (CCTA) has the ability to visualize both the coronary artery lumen and any surrounding atherosclerotic plaque.^3,4^ This allows additional assessment of coronary plaque burden and plaque type, both of which may improve our understanding of coronary atherosclerosis and cardiovascular risk prediction.^5,6^

Coronary artery calcium scoring on noncontrast computed tomography (CT) using the Agatston method^7^ has been widely used as a surrogate marker of coronary plaque burden, providing powerful cardiovascular risk prediction^8,9^ that is additive to traditional cardiovascular risk scores.^10,11^ However, these macrocalcified plaques are generally considered a more stable form of atherosclerosis and, hence, relatively unlikely to rupture and cause myocardial infarction directly.^12^ There is considerable interest in quantifying the burden of less stable plaque components that might more directly contribute to events.

Histological plaque characteristics that have been associated with plaque rupture and myocardial infarction include inflammation, microcalcification, a thin fibrous cap, and a large lipid-rich necrotic core.^13–15^ On CCTA, a lipid rich necrotic core can be detected as low-attenuation noncalcified plaque.^16^ We have recently demonstrated that visually identifying such plaque may be of benefit in predicting the future risk of myocardial infarction.^17^ However, such qualitative assessments are relatively subjective, time consuming, and nonquantitative. Recent developments now allow the reproducible and semiautomatic quantification of the burden of calcified, noncalcified, and low-attenuation plaque across the coronary arteries on contrast CT angiography.^18,19^

This study aimed to investigate whether quantification of CCTA-defined low-attenuation plaque improves the prediction of fatal or nonfatal myocardial infarction compared with cardiovascular risk scores, Agatston coronary artery calcium scoring, and the severity of obstructive coronary artery disease in stable patients with chest pain undergoing CCTA.

## Methods

### Study Design

The data supporting the findings of this study are available from the corresponding author for checking the reproducibility of the study results. The SCOT-HEART multicenter randomized controlled trial (Scottish Computed Tomography of the HEART) assessed the use of CCTA in patients with suspected angina attributable to coronary artery disease (ClinicalTrials.gov. Unique identifier: NCT01149590).^20^ The study was approved by the local ethics committee and written informed consent was provided by all participants. The primary results have been published previously.^21–23^ Patients attending cardiology outpatient clinics were randomized to standard care or standard care plus CT. Of the 4146 trial participants, 2073 were randomized to undergo CT. Of these, 1778 underwent CT and 1769 scans were available and of suitable quality for analysis in this substudy. Cardiovascular risk was calculated using the ASSIGN (Assessing cardiovascular risk using SIGN guidelines) cardiovascular risk score.^24^ This is a well-validated risk score based on the Framingham risk score and has been calibrated to the Scottish population.

### CT Imaging

Participants underwent both noncontrast electrocardiogram-gated CT for calcium scoring and contrast enhanced electrocardiogram-gated CCTA.^20^ Imaging was performed at one of three sites using either 64- or 320-detector row scanners (Brilliance 64, Philips Medical Systems, Netherlands; Biograph mCT, Siemens, Germany; Aquilion ONE, Toshiba Medical Systems, Japan). Tube current, voltage, and volume of iodine-based contrast were adjusted based on body mass index.

### Visual Assessment of CT

Coronary artery calcium score and coronary artery stenosis assessments were obtained from the SCOT-HEART database. Coronary artery calcium score was assessed using the Agatston scoring method, as described previously.^7,20^ Coronary artery stenoses were visually assessed on a 15-segment basis by trained observers with excellent inter- and intraobserver variability, as described previously.^25^ In brief, the luminal area stenosis was here visually classified as normal, nonobstructive (<50% in left main stem or <70% in other segments), and obstructive (≥50% in left main stem or ≥70% in other segments). Obstructive coronary artery disease was defined as the presence of 1 or more major epicardial vessel with an obstructive stenosis.

### Quantitative Assessment of CT

Quantitative assessment of atherosclerotic plaque subtypes was performed using standardized semiautomatic software (Autoplaque, Version 2.5, Cedars-Sinai Medical Center) by 1 of 4 trained observers. Excellent interobserver, intraobserver, and interscan agreement has previously been established for this technique (Spearman *r*=0.87–0.99).^18,26^ Quantitative analysis was performed for all patients who had 1 or more segments of nonobstructive or obstructive disease or a coronary artery calcium score greater than 0. For patients with normal coronary arteries, the plaque volumes and burden were set to 0.

**Figure 1.**
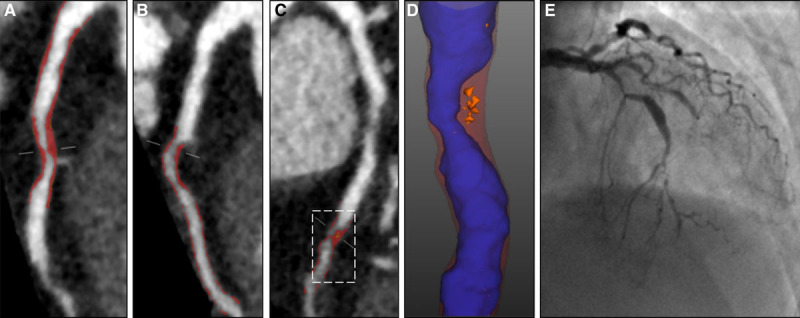
**Coronary CT angiography (CCTA) plaque analysis.** Images from a 67-year-old female who presented with atypical chest pain. She was a nonsmoker with a history of hypertension, a previous transient ischemic attack, and normal exercise tolerance test. Her 10-year cardiovascular risk score was 14%, coronary artery calcium score was 62 Agatston units, and coronary computed tomography angiography identified obstructive disease in the left anterior descending (LAD) and first diagonal. Curved planar reformations show (**A**) proximal LAD, (**B**) first diagonal, and (**C**) mid-LAD. Red overlay illustrates noncalcified plaque in the individual segment only, but all plaque in each vessel was analyzed for the per patient assessment. **D**, Shows a zoomed in view of the mid LAD plaque with blue lumen, red noncalcified plaque and orange low-attenuation plaque. She subsequently presented with acute myocardial infarction and underwent invasive coronary angiography (**E**).

Coronary artery centerlines were extracted using a semiautomated method. A circular region of interest was placed in the proximal aorta to define the normal reference blood pool attenuation. The proximal and distal aspects of coronary artery segments with atherosclerotic plaque were manually defined. Scan-specific thresholds for plaque constituents were generated, as described previously.^27^ Vessel lumen, wall, and plaque constituents were automatically identified, with manual adjustment performed as required (Figure 1). Automatic quantification of area stenosis at the narrowest point of the segmented lumen was performed.

Plaque volumes (in mm^3^) were measured for the following plaque subtypes: total plaque, calcified plaque, noncalcified plaque and low-attenuation plaque (defined by an attenuation of <30 Hounsfield units [HU]).^19^ Plaque burden (as a percentage) was calculated for each of the total plaque, noncalcified plaque, low-attenuation plaque, and calcified plaque, by dividing the relevant plaque volume by the vessel volume of the region assessed, multiplying by 100, and summing on a per patient basis.^19^ Plaque burden was used as our primary assessment for each plaque type.

### Outcomes

The primary event for this substudy was the occurrence of fatal or nonfatal myocardial infarction. Information on cardiovascular events and mortality was obtained from the electronic Data Research and Innovation Service of the National Health Services Scotland and were confirmed by review of the patient’s electronic health records where required. Follow-up was administratively censored as of January 1, 2018, for patients without an event. Scottish health record linkage has previously been shown to have an excellent correlation (>95%) for accurately identifying clinical events recorded and adjudicated as part of a regulated clinical trial.^28^

### Statistical Analysis

Statistical analysis was performed using R, version 3.5.0 (R Foundation for Statistical Computing, Vienna, Austria). Quantitative data are presented as mean and standard deviation or, if not normally distributed, as median and interquartile interval (IQI). Statistical significance was assessed using Pearson chi-square test, Fisher exact test, Student *t* test, Kruskal–Wallis test, or Mann–Whitney *U* test as appropriate. Correlations for variables that were not normally distributed were assessed using Spearman rank order correlation. Correlation coefficients of <0.2 were regarded as very weak, 0.2 to <0.40 as weak, 0.40 to <0.60 as moderate, 0.6 to <0.80 as strong, and 0.8 to 1 as very strong. Outcome data were analyzed using Cox proportional hazards regression and presented graphically using cumulative incidence plots using the Kaplan–Meier method. Hazard ratios (HR) and 95% CI were calculated from the Cox model. Deaths not classified as coronary heart disease deaths were censored for both the Cox regression analysis and the cumulative incidence plots. Univariable analysis was performed for all quantified plaque parameters. Multivariable models were constructed including the individual plaque parameters and Agatston coronary artery calcium score. Coronary artery calcium score and plaque burdens were log transformed for analysis. Receiver operating characteristic (ROC) curve analysis was performed to identify the optimum cut-off for low-attenuation plaque burden to identify patients at increased risk of coronary heart disease death or nonfatal myocardial infarction using Youden J statistic. A statistically significant difference was defined as a 2-sided *P* value <0.05. The imaging data that support the findings of this study are available from the corresponding author upon reasonable request.

## Results

### Patient Population

This substudy included all 1769 patients where CCTA images were of suitable image quality for analysis. The mean age of these patients was 58±10 years, 56% were male, and the mean 10-year cardiovascular risk score was 18±11% (Table 1). The median coronary artery calcium score was 21 (IQI, 0–230) Agatston units. Normal coronary arteries were identified in 646 patients (37%), nonobstructive coronary artery disease in 671 patients (38%) and obstructive coronary artery disease in 452 patients (26%). The median total plaque burden for all patients (Table 2) was 39% (IQI, 0–49), with noncalcified plaque burden 36% (IQI, 0–46), low-attenuation plaque burden 4.2% (IQI, 0–6.9), and calcified plaque burden 0.4% (IQI, 0–2.8).

**Table 1.**
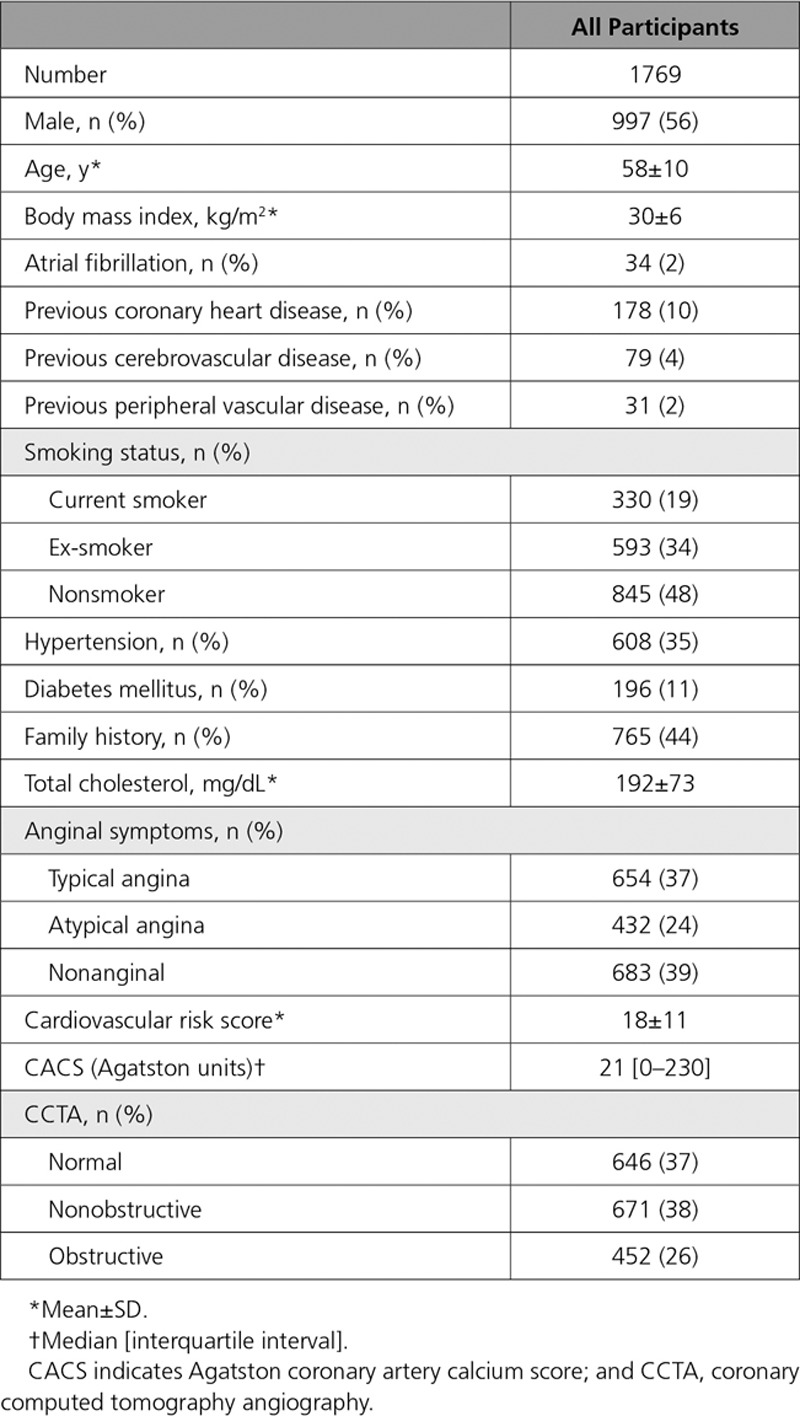
Study Population

**Table 2.**
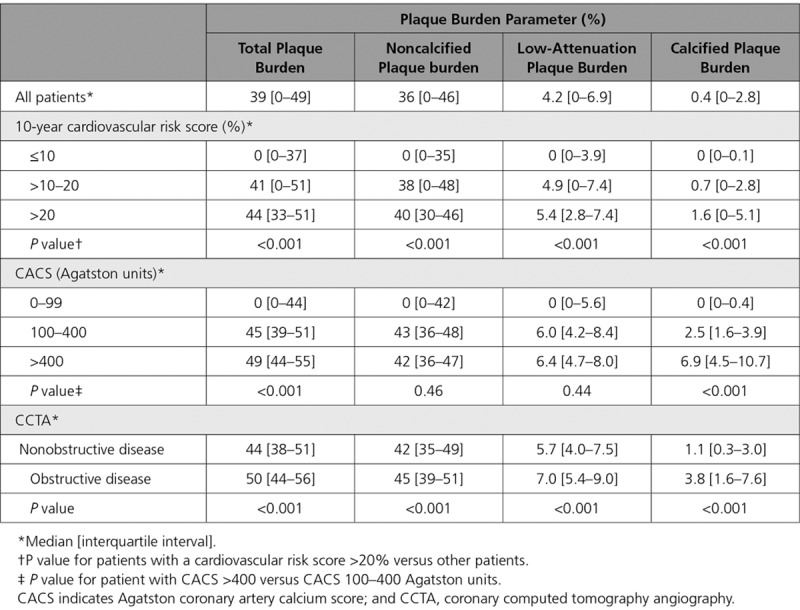
Quantitative Plaque Burden in Patients With Different Cardiovascular Risk Scores, Coronary Artery Calcification, and CCTA Findings

### Cardiovascular Risk Score and Plaque Type

**Figure 2.**
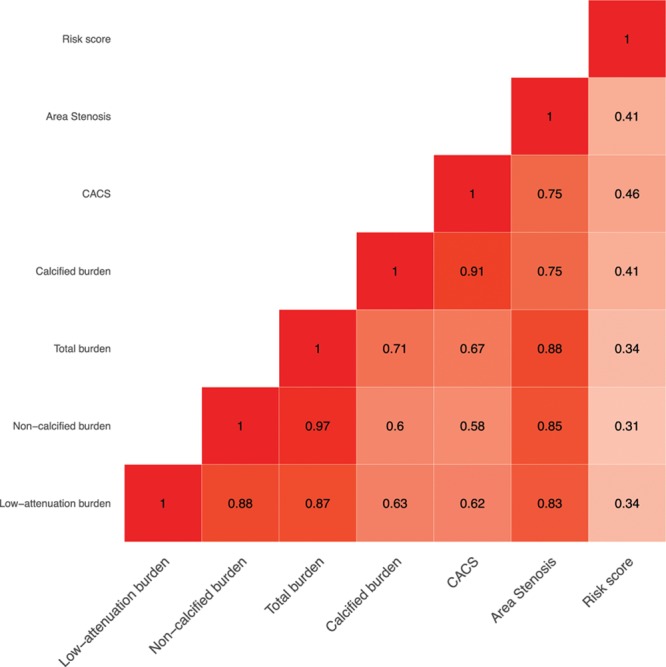
**Correlations between plaque burden subtypes, calcium score, coronary stenosis and cardiovascular risk score.** Correlations between plaque burden subtypes and Agatston coronary artery calcium score, coronary artery area stenosis and ASSIGN (Assessing cardiovascular risk using SIGN guidelines) cardiovascular risk score. *P*<0.001 for all. CACS indicates Agatston coronary artery calcium score.

**Figure 3.**
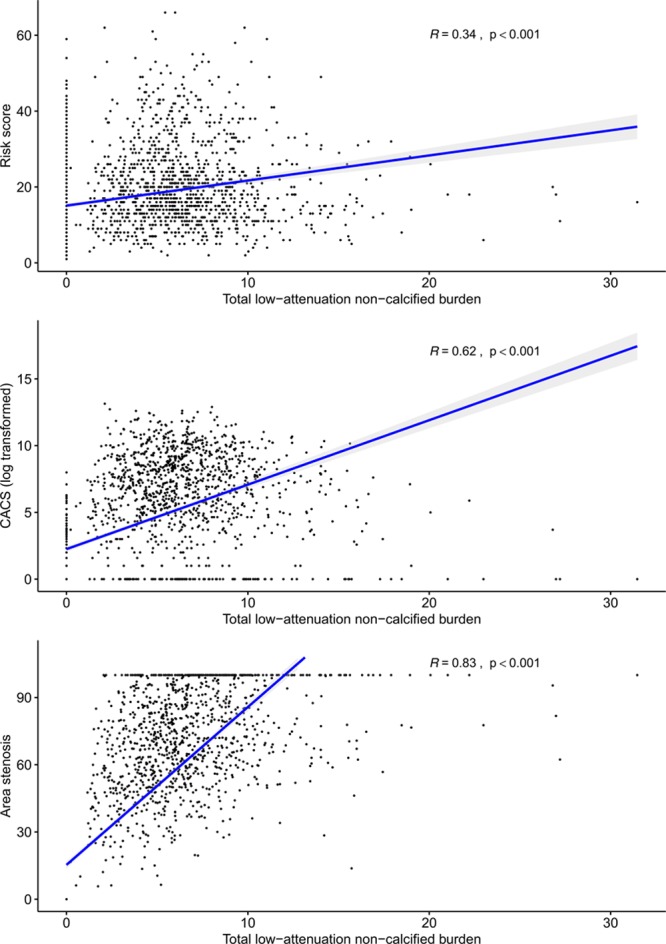
**Correlations between low-attenuation plaque burden, cardiovascular risk score, calcium score and coronary stenosis.** Correlations between total low-attenuation plaque burden and 10-year cardiovascular risk score, Agatston coronary artery calcium score, and coronary artery area stenosis. CACS indicates Agatston coronary artery calcium score.

Compared with those with a cardiovascular risk score ≤10, patients with a cardiovascular risk score >10 had an increase in all subtypes of plaque burden (*P*<0.001 for all) (Table 2). Similarly, patients with a cardiovascular risk score >20 had a higher low-attenuation plaque burden, total plaque burden, and calcified plaque than patients with a score ≤20. There was a weak correlation between cardiovascular risk scores and total plaque burden (*r*=0.34; *P*<0.001) (Figure 2), and weak to moderate correlations with plaque burden subtypes including low-attenuation plaque (*r*=0.34; *P*<0.001) (Figure 2 and 3).

### Coronary Artery Calcium Score and Plaque Type

A very strong correlation was observed between the Agatston coronary artery calcium score and the calcified plaque burden quantified on CCTA (*r*=0.91; *P*<0.001) (Figure 2). Moderate to strong correlations were observed between the Agatston coronary artery calcium score and other plaque subtypes, including the total plaque burden (*r*=0.67; *P*<0.001), noncalcified plaque burden (*r*=0.58; *P*<0.001), and low-attenuation plaque burden (*r*=0.62; *P*<0.001) (Figure 3). Low-attenuation plaque burden demonstrated very strong correlations with the total plaque burden (*r*=0.87; *P*<0.001) and noncalcified plaque burden (*r*=0.88; *P*<0.001), and a moderate to strong correlation with the calcified plaque burden (*r*=0.63; *P*<0.001). Patients with an Agatston coronary artery calcium score greater than 400 had a higher total plaque burden and calcified plaque burden than patients with a score less than 400 (*P*<0.001). The low-attenuation plaque burden was numerically higher but this was not statistically significant (Table 2).

### Coronary Artery Stenosis and Plaque Type

Patients with visually assessed obstructive coronary artery disease had higher total plaque burden, calcified plaque burden, noncalcified plaque burden and low-attenuation plaque burden compared with patients with nonobstructive coronary artery disease (Table 2). Moreover, for patients with 1-, 2-, and 3-vessel disease, there was a stepwise increase in the total plaque burden, low-attenuation plaque burden and calcified plaque burden (Table I in the online-only Data Supplement).

### Clinical Events

**Figure 4.**
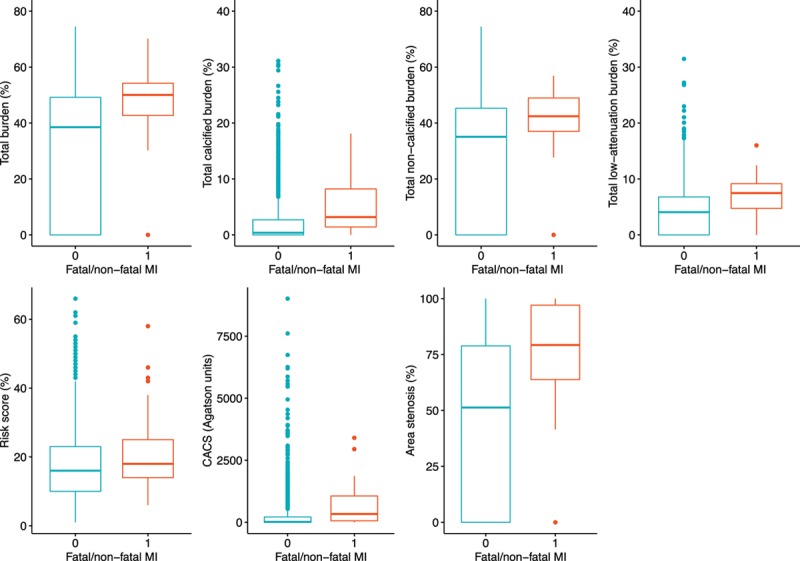
**Plaque burden and fatal or nonfatal myocardial infarction.** Quantitative assessment of atherosclerotic plaque burden in patients with and without a primary event of fatal or nonfatal myocardial infarction (*P*≤0.01 for all). CACS indicates Agatston coronary artery calcium score, and MI, myocardial infarction.

Over a median follow-up of 4.7 years (IQI, 4.0–5.7), the primary event point of fatal or nonfatal myocardial infarction occurred in 41 patients (2.3%). Patients who suffered a fatal or nonfatal myocardial infarction had higher total plaque, noncalcified plaque, low-attenuation plaque and calcified plaque burden (Table 3; Figure 4). In univariable analysis, the total plaque, noncalcified plaque, low-attenuation plaque and calcified plaque burdens were all associated with an increased risk of fatal or nonfatal myocardial infarction (Table 4; Table II in the online-only Data Supplement). In multivariable analysis, the low-attenuation plaque burden was the strongest predictor of the primary event (HR, 1.60 [95% CI, 1.10–2.34] per doubling; *P*=0.014), adding incremental value to the cardiovascular risk score (HR, 1.00 [95% CI, 0.98–1.03]; *P*=0.821), coronary artery calcium score (HR, 1.13 [95% CI, 1.01–1.27]; *P*=0.041), and the presence of visually observed obstructive coronary artery disease (HR, 1.20 [95% CI, 0.58–2.48]; *P*=0.621) (Table 4; Table III in the online-only Data Supplement).

**Table 3.**
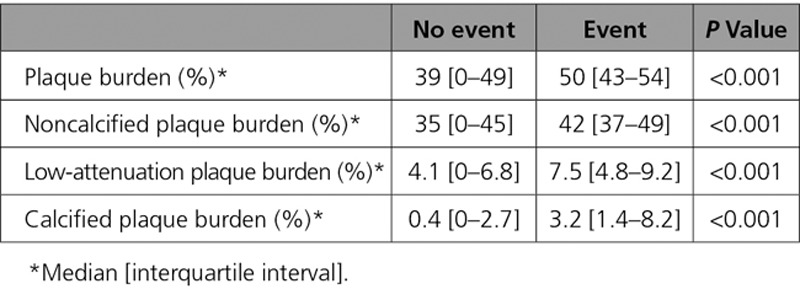
Quantitative Assessment of Plaque in Patients With and Without a Primary Event of Coronary Heart Disease Death or Nonfatal Myocardial Infarction

**Table 4.**
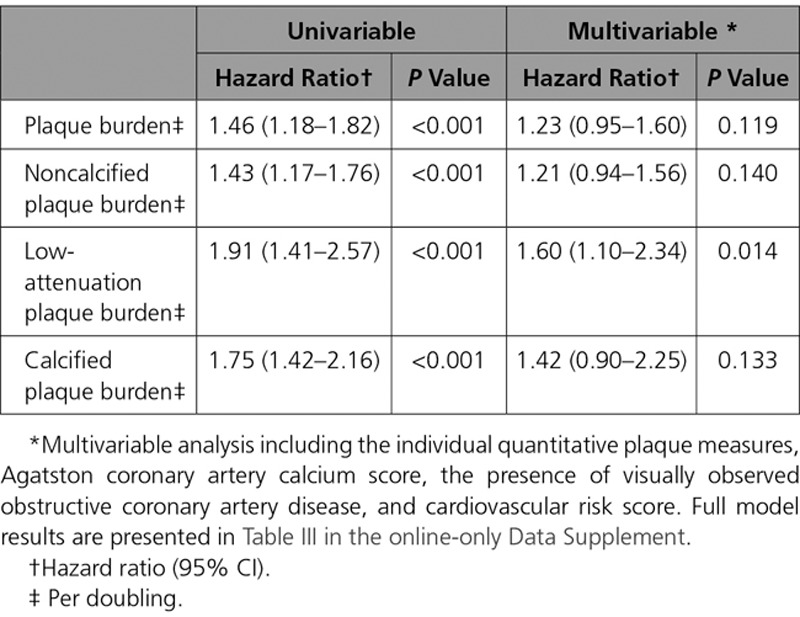
Univariable and Multivariable Analysis for Plaque Subtypes and the Primary End Point of Fatal or Nonfatal Myocardial Infarction

**Figure 5.**
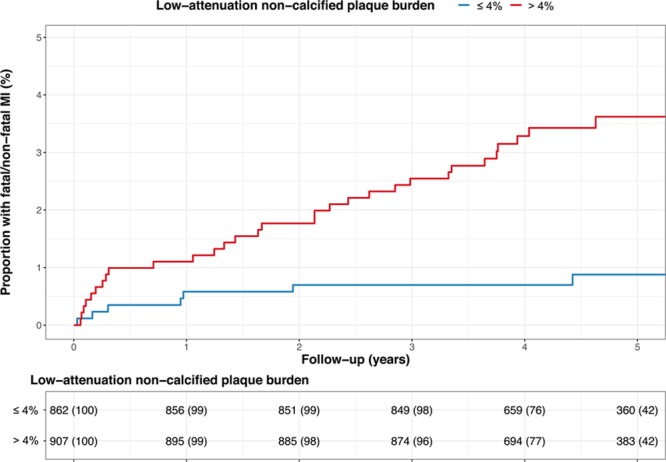
**Low-attenuation plaque burden and fatal or nonfatal myocardial infarction.** Cumulative incidence of fatal or nonfatal myocardial infarction in patients with and without a low-attenuation plaque burden greater than 4%. MI indicates myocardial infarction.

Receiver operating characteristic curve analysis identified that the optimum threshold for low-attenuation plaque burden was 4%. Patients with a low-attenuation plaque burden above this threshold were nearly 5 times more likely to suffer a fatal or nonfatal myocardial infarction (HR, 4.65 [95% CI, 2.06–10.5]; *P*<0.001) (Figure 5). This discriminatory ability was particularly important in patients with nonobstructive coronary artery disease (Table IV and Figures I and II in the online-only Data Supplement). Patients with nonobstructive coronary artery disease and low-attenuation plaque burden >4% were more than 6 times more likely to suffer a fatal or nonfatal myocardial infarction compared with patients with normal coronary arteries (HR, 6.61 [95% CI, 1.91 to 22.82]; *P*=0.003), but patients with nonobstructive disease and a low-attenuation plaque burden ≤4% were not at a significantly increased risk (HR, 1.33 [95% CI, 0.14–12.77]; *P*=0.806).

## Discussion

In this large multicenter study, we have shown that quantitative assessment of atherosclerotic plaque can identify patients who are at an increased risk of coronary events. Low-attenuation plaque burden was the strongest predictor of outcome, with patients with a low-attenuation plaque burden >4% 5 times more likely to suffer a fatal or nonfatal myocardial infarction. Despite collinearity and strong correlations, this association remained irrespective of cardiovascular risk score, Agatston coronary artery calcium score or the presence of obstructive coronary artery disease. We conclude that low-attenuation plaque burden is the best predictor of clinical outcome, and that this underscores the dominant nature of plaque burden and plaque type in the pathogenesis of myocardial infarction.

Markers of plaque burden provide powerful prediction of cardiovascular events on the basis that the more plaque a patient has, the more likely an individual lesion will rupture and cause myocardial infarction. However, plaque type is also important, with plaque rupture most commonly associated with inflamed plaques with a thin fibrous cap and large necrotic core.^14^ The latter can be detected on CT as low-attenuation plaque, although it has previously been challenging to quantify the burden of low-attenuation plaque across the coronary vasculature. This is now possible in a rapid and robust fashion with semiautomatic software. In this study, we have demonstrated that low-attenuation plaque provides more powerful prediction of clinical events than cardiovascular risk score, Agatston coronary artery calcium scoring, or the presence and severity of obstructive coronary artery disease. Indeed, our data would suggest that these other markers are associated with clinical events by virtue of their association with low-attenuation plaque burden, and that it is preferable to measure low-attenuation plaque directly for optimum patient risk stratification.

This study provides further evidence of the limitations of cardiovascular risk scores in the prediction of cardiac events. Cardiovascular risk scores are widely used in clinical practice to assess the requirement for preventative therapy,^29^ and play a central role in the management pathways for patients with suspected coronary artery disease, in both US and European guidelines.^1,2^ In this study, cardiovascular risk score demonstrated only moderate correlation with low-attenuation plaque burden. Such scores are therefore limited in their ability to identify patients with the atherosclerotic plaque at risk of subsequent events, perhaps explaining their poor prognostic performance here and in other studies.^30^

Coronary artery calcification is widely used as a marker of coronary artery disease and is associated with an increased risk of subsequent cardiac events.^9,17,31^ In this study, we have shown that quantification of calcification on CCTA correlates well with the Agatston coronary artery calcium score performed on noncontrast CT, highlighting that there is limited value to additional noncontrast imaging when CCTA is performed. However, similar to the cardiovascular risk score, assessment of coronary artery calcification only provides risk by association, quantifying stable calcified plaques rather than the disease that causes subsequent cardiac events. When the Agatston coronary artery calcium score was first established, CCTA had not been developed for routine clinical use, and the calcium score was a useful surrogate for the presence of coronary artery disease. Indeed, we have previously reported that it is a strong predictor of outcome, outperforming qualitative assessments of adverse coronary artery plaque.^17^ However, quantitative CCTA analysis now provides assessment of coronary plaque burden, with the added ability of more accurately quantifying the plaque type most closely associated with events.

The identification of obstructive coronary artery stenosis is central to the diagnosis and management approach of patients presenting with chest pain. It is an established and powerful predictor of an increased risk of subsequent cardiac events which is embedded within clinical practice. It is a central tenet of cardiology that this risk increases with the number of vessels involved.^5,32^ However, most coronary events occur in segments that do not have obstructive stenoses on preceding imaging,^33–35^ and percutaneous coronary intervention on coronary artery stenoses does not prevent or reduce subsequent rates of myocardial infarction.^36^ This study provides support for the hypothesis that obstructive disease is indirectly associated with subsequent outcomes because of its correlation with plaque burden. Indeed, we observed a stepwise increased in plaque burden, including low-attenuation plaque, on moving from patients with nonobstructive coronary artery disease to those with 1-, 2- and 3-vessel obstructive coronary artery disease. Therefore, similar to the cardiovascular risk scores and calcium scoring, we propose that the predictive ability of obstructive coronary artery stenoses is, at least in part, attributable to its association with the low-attenuation plaque.

Quantitative assessment of plaque can provide standardized assessment with good observer variability and correlation with intravascular ultrasound.^27^ Previous studies have identified that CCTA plaque burden is associated with subsequent outcomes, including the ICONIC (Incident Coronary Syndromes Identified by Computed Tomography) substudy of the CONFIRM (Coronary CT Angiography Evaluation for Clinical Outcomes: An International Multicenter) registry^37^ and the PARADIGM (Progression of Atherosclerotic Plaque Determined by Computed Tomographic Angiography Imaging) registry.^38^ Low-attenuation plaque in particular has been associated with an increased risk.^39–41^ This is in keeping with our data and the concept that plaques with large necrotic cores are more prone to rupture than more stable calcified plaque subtypes.

This study has several limitations. It used a single technique to analyze plaque. Further investigation and external validation are required for this to become accepted and part of clinical practice and these should also incorporate analysis from more than one type of scanner or reconstruction algorithm. Although several parts of the analysis performed in this study were automated, the assessment of plaque type across the entire coronary tree takes 20 to 30 minutes.^41^ Future software incorporating further automation and machine learning techniques, would therefore help facilitate more widespread clinical adoption. In addition, further quantified parameters such as pericoronary adipose tissue attenuation, radiomic analyses, or positron emission tomography features may provide additional information on cardiovascular risk in addition to the plaque parameters assessed in this study.^12,42,43^ Finally, in the SCOT-HEART trial, treatment was instigated based on CCTA findings, and we cannot exclude the possibility that these treatment decisions may have influenced our findings. However, we would suggest that such interventions are likely to make our estimates of the association and effect sizes conservative.

In conclusion, this large multicenter study shows that an increased burden of low-attenuation plaque is the principal predictor of increased coronary events, above and beyond other established classic markers of cardiovascular risk, including coronary artery stenosis severity.

## Sources of Funding

This trial was funded by The Chief Scientist Office of the Scottish Government Health and Social Care Directorates (CZH/4/588), with supplementary awards from Edinburgh and Lothian’s Health Foundation Trust and the Heart Diseases Research Fund. M.C.W. (FS/11/014 and CH/09/002), D.E.N. (CH/09/002, RG/16/10/32375, RE/18/5/34216), and M.R.D. (FS/14/78/31020) are supported by the British Heart Foundation. M.C.W. was supported by The Chief Scientist Office of the Scottish Government Health (PCL/17/04). D.E.N. is the recipient of a Wellcome Trust Senior Investigator Award (WT103782AIA). E.J.R.v.B. is supported by the Scottish Imaging Network: A Platform of Scientific Excellence (SINAPSE). P.D.A. is supported by a National Heart Foundation of New Zealand Senior Fellowship (1844). M.R.D. is supported by the Sir Jules Thorn Biomedical Research Award 2015 (15/JTA). The Royal Bank of Scotland supported the provision of 320-multidetector CT for NHS Lothian and the University of Edinburgh. The Edinburgh Imaging facility QMRI (Edinburgh) is supported by the National Health Service Research Scotland (NRS) through National Health Service Lothian Health Board. The Clinical Research Facility Glasgow and Clinical Research Facility Tayside are supported by National Health Service Research Scotland (NRS). P.M. and D.D. are supported by National Institute of Health/National Heart, Lung, and Blood Institute grant 1R01HL133616. This work is supported in part by National Institute of Health/National Heart, Lung, and Blood Institute grant 1R01HL133616 and the Miriam and Sheldon G. Adelson Medical Research Foundation.

## Disclosures

D.D., S.C., P.S., and D.S.B. received software royalties from Cedars-Sinai Medical Center. D.D., P.S., and D.S.B. hold a patent (US8885905B2 in U.S. and WO patent WO2011069120A1, Method and System for Plaque Characterization).

## Supplementary Material


