# Supplementary material for: Low-Attenuation Noncalcified Plaque on Coronary Computed Tomography Angiography Predicts Myocardial Infarction: Results From the Multicenter SCOT-HEART Trial (Scottish Computed Tomography of the HEART)
Source: Circulation. 2020 Mar 16;141(18):1452–62. doi: 10.1161/CIRCULATIONAHA.119.044720 (PMC7195857; doi:10.1161/CIRCULATIONAHA.119.044720)
Supplement: Supplementary file 1 [file cir-141-1452-s001.pdf]

SUPPLEMENTAL MATERIAL

Supplemental Tables

Supplemental Table 1: Quantitative plaque burden in patients 1, 2 and 3 or more vessels of obstructive coronary artery disease.

	**No vessels with obstructive CAD**	**1-Vessel obstructive CAD**	**2-Vessel obstructive CAD**	**3-Vessel obstructive CAD**	**P**
Plaque burden (%)	26 [0 to 44]	48 [43 to 55]	51 [45 to 58]	52 [47 to 58]	**<0.001**
Non-calcified plaque burden (%)	24 [0 to 42]	44 [39 to 50]	45 [38 to 52]	46 [39 to 49]	**<0.001**
Low-attenuation plaque burden (%)	1.5 [0 to 5.8]	6.4 [4.7 to 8.6]	7.2 [5.4 to 8.8]	7.7 [6.0 to 9.5]	**<0.001**
Calcified plaque burden (%)	0 [0 to 1.2]	2.3 [0.8 to 5.7]	4.4 [2.1 to 7.5]	6.1 [3.4 to 11.3]	**<0.001**

Median [interquartile range].
CAD, coronary artery disease.

Supplemental Table 2: Univariable analysis for plaque subtypes and the primary end-point of fatal or non-fatal myocardial infarction.

	**No Event**	**Event**	**Univariable**	
			**Hazard ratio**	**P**
Agatston CACS (Agatston Units)	19 [0 to 217]	336 [62 to 1064]	**1.23 (1.13 to 1.35) ***	**<0.001**
Obstructive disease	430 (25%)	22 (54%)	**3.35 (1.81 to 6.19)**	**<0.001**
Cardiovascular risk score (%)	18 ± 11	22 ± 12	1.03 (1.00 to 1.05)	0.024

Hazard ratio (95% confidence interval). Median [Interquartile range]

* Per doubling

CACS, coronary artery calcium score.

Supplemental Table 3: Multivariable models for the primary end-point of fatal or non-fatal myocardial infarction.

		**Hazard ratio**	**P**
Model 1	Plaque burden *	1.23 (0.95 to 1.60)	0.119
	Agatston CACS *	1.12 (0.98 to 1.27)	0.089
	Obstructive disease †	1.37 (0.66 to 2.84)	0.398
	Cardiovascular risk score	1.00 (0.98 to 1.03)	0.828
Model 2	Non-calcified plaque burden *	1.21 (0.94 to 1.56)	0.140
	Agatston CACS *	1.13 (1.00 to 1.28)	0.056
	Obstructive disease †	1.37 (0.66 to 2.84)	0.396
	Cardiovascular risk score	1.00 (0.98 to 1.03)	0.831
Model 3	Low-attenuation plaque burden *	**1.60 (1.10 to 2.34)**	**0.014**
	Agatston CACS *	**1.13 (1.01 to 1.27)**	**0.041**
	Obstructive disease †	1.20 (0.58 to 2.48)	0.621
	Cardiovascular risk score	1.00 (0.98 to 1.03)	0.821
Model 4	Calcified plaque burden *	1.42 (0.90 to 2.25)	0.133
	Agatston CACS *	1.06 (0.88 to 1.28)	0.549
	Obstructive disease †	1.39 (0.64 to 3.01)	0.400
	Cardiovascular risk score	1.01 (0.98 to 1.04)	0.694
Model 5	Plaque burden *	0.38 (0.01 to 18.92)	0.629
	Non-calcified plaque burden *	2.02 (0.04 to 94.24)	0.720
	Low-attenuation plaque burden *	**2.25 (1.17 to 4.34)**	**0.015**
	Calcified plaque burden *	**1.78 (1.02 to 3.10)**	**0.042**
Model 6	Plaque burden *	0.39 (0.01 to 21.9)	0.645
	Non-calcified plaque burden *	2.01 (0.04 to 109.7)	0.732
	Low-attenuation plaque burden *	**2.22 (1.13 to 4.35)**	**0.020**
	Calcified plaque burden *	1.79 (0.85 to 3.77)	0.126
	Agatston CACS *	0.98 (0.80 to 1.21)	0.881
	Obstructive disease †	1.10 (0.52 to 2.32)	0.802
	Cardiovascular risk score	1.01 (0.98 to 1.03)	0.702

Hazard ratio (95% confidence interval).
* Per doubling.
† Presence of obstructive coronary artery disease on visual assessment
CACS, coronary artery calcium score.

Supplemental Table 4: Univariable analysis for plaque subtypes and the primary end-point of fatal or non-fatal myocardial infarction compared to patients with normal coronary arteries.

	**Hazard ratio**	**P**
Non-obstructive disease with LA plaque <4%	1.33 (0.14 to 12.77)	0.806
Non-obstructive disease with LA plaque >4%	**6.61 (1.91 to 22.82)**	**0.003**
Obstructive disease	**10.56 (3.16 to 35.30)**	**<0.001**

LA, low-attenuation non-calcified plaque.

Supplemental Figures and Figure Legends

Supplemental Figure 1: Cumulative incidence of fatal or non-fatal myocardial infarction (MI) in patients with normal coronary arteries, obstructive coronary artery disease, and non-obstructive coronary artery disease with and without low-attenuation non-calcified plaque burden above 4%.

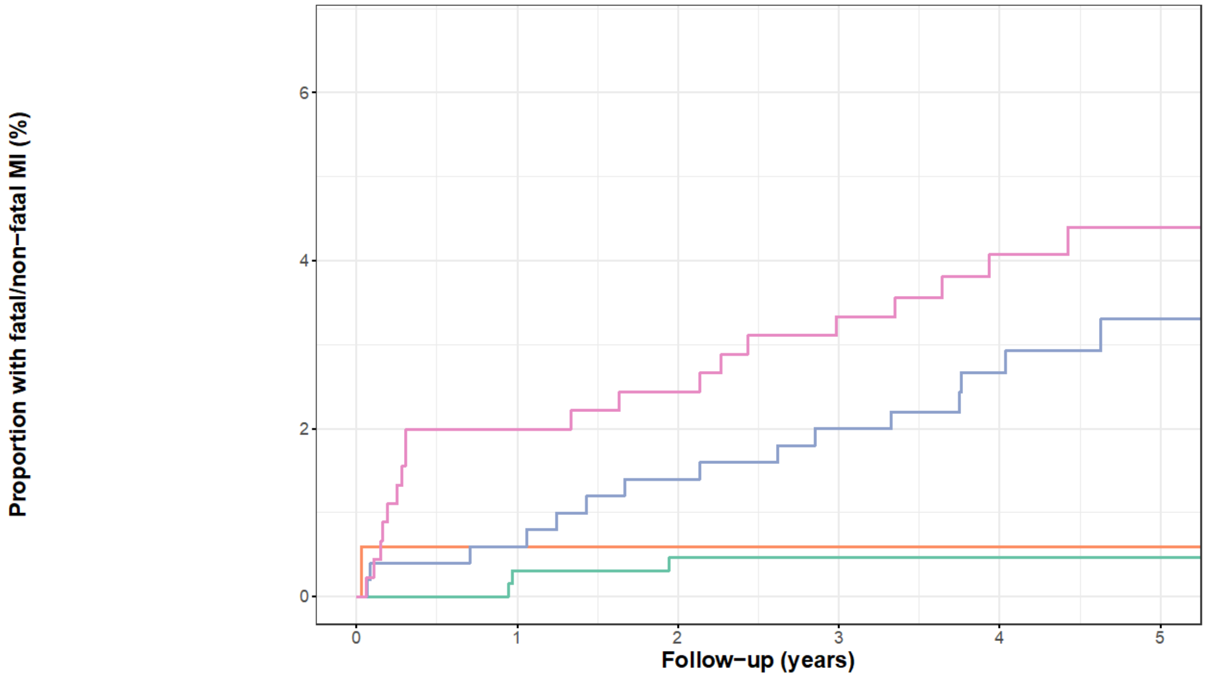

Normal	646 (100)	644 (100)	641 (99)	640 (99)	502 (78)	288 (45)
Non obstructive, LA<=4%	168 (100)	167 (99)	165 (98)	165 (98)	123 (73)	54 (32)
Non obstructive, LA>4%	503 (100)	498 (99)	493 (98)	489 (97)	380 (76)	188 (37)
Obstructive	452 (100)	442 (98)	437 (97)	429 (95)	348 (77)	213 (47)

LA, low attenuation non-calcified plaque; MI, myocardial infarction.

Supplemental Figure 2: Cumulative incidence of fatal or non-fatal myocardial infarction in patients with no low-attenuation plaque and low-attenuation plaque burden above 4%.

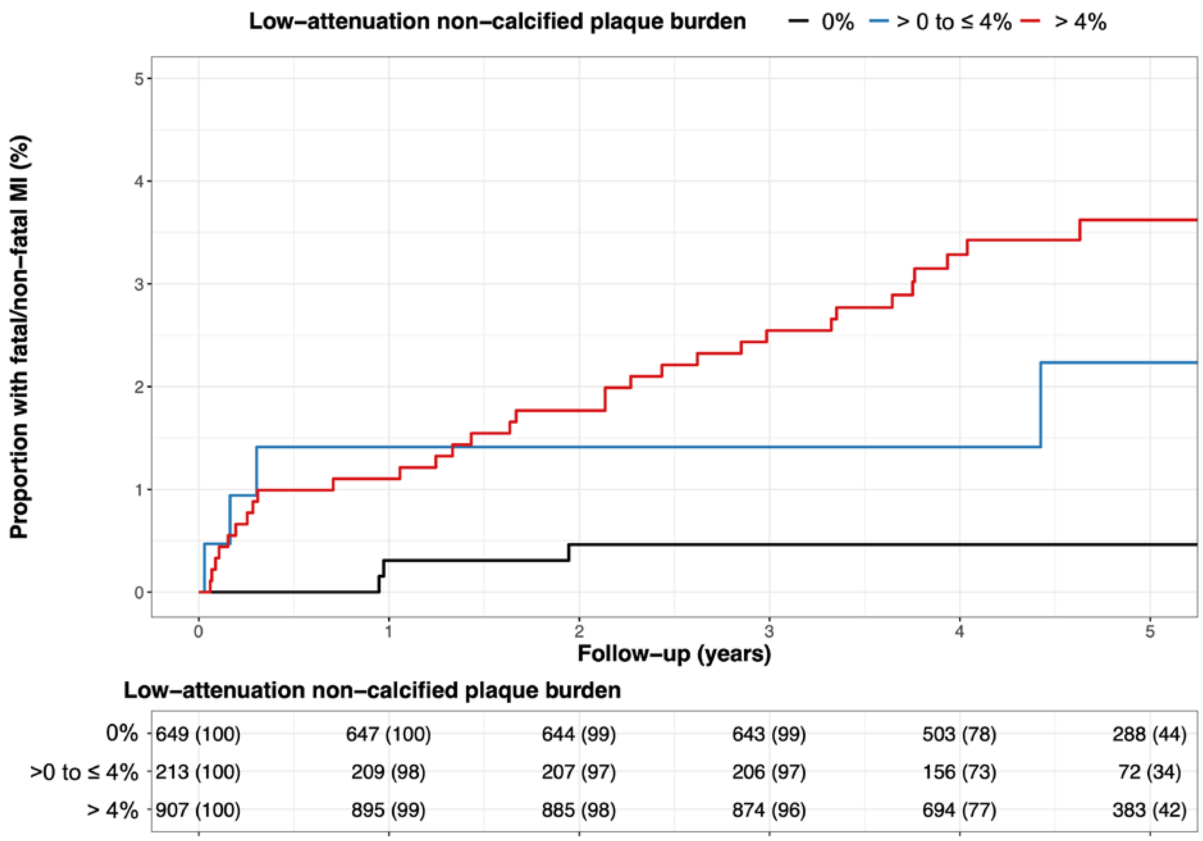

MI, myocardial infarction.
